# The Role of Physical Activity Opportunities and Local Authority Engagement in Promoting Healthy Living and Increasing Life Expectancy

**DOI:** 10.3390/healthcare13172110

**Published:** 2025-08-25

**Authors:** Mădălina Giorgiana Mangra, Gabriel Ioan Mangra, Claudiu George Bocean, Anca Antoaneta Vărzaru, Cristina Claudia Rotea, Constantin-Cristian Văduva

**Affiliations:** 1 Department of Finance, Banking and Economic Analysis, Faculty of Economics and Business Administration, University of Craiova, 200585 Craiova, Romania; madalina.mangra@edu.ucv.ro; 2 Department of Theory and Methodology of Motor Activities, University of Craiova, 200585 Craiova, Romania; 3 Department of Management, Marketing and Business Administration, Faculty of Economics and Business Administration, University of Craiova, 200585 Craiova, Romania; 4 Department of Economics, Accounting and International Business, Faculty of Economics and Business Administration, University of Craiova, 200585 Craiova, Romania; anca.varzaru@edu.ucv.ro; 5 Faculty of Mechanics, University of Craiova, 200585 Craiova, Romania; cristina.rotea@edu.ucv.ro; 6 Department of Obstetrics and Gynecology, University of Medicine and Pharmacy, 200349 Craiova, Romania; cristian.vaduva@umfcv.ro; 7 Clinic of Obstetrics and Gynecology, Filantropia Clinical Hospital, 200143 Craiova, Romania; 8 Department of Obstetrics, Gynecology and IVF, HitMed Medical Center, 200130 Craiova, Romania

**Keywords:** physical activity, exercise or play sports, local providers, local authorities, healthy life years, life expectancy

## Abstract

**Background**: Physical activity is essential for public health, yet disparities in access to exercise opportunities and institutional support remain significant across European regions. **Objectives**: This study examines how locally available physical activity options and support from local authorities relate to exercise participation, life expectancy, and healthy life years within the European Union. **Methods**: Using artificial neural networks and cluster analysis, the research identifies patterns across EU countries and explores associations between exercise behaviors and public health outcomes. **Results**: The MLP analysis showed that moderate regular physical activity had the most decisive influence on predicting healthy life years (100%), followed by regular activity (44.7%). In comparison, inactivity had a notable negative impact (40.5%). Life expectancy was most closely associated with the model’s strongest predictive pathway (weight = 2.395). Access to physical activity opportunities (100%) and the presence of supportive community providers (90.8%) were the most influential factors in encouraging active lifestyles. **Conclusions:** Populations with greater access and engagement in physical activity tend to enjoy longer life expectancies and more years of good health. While the study’s observational nature limits causal interpretations, the findings highlight the potential of community-level strategies and infrastructural investment to foster active lifestyles and enhance population health across varied local contexts.

## 1. Introduction

Physical activity (PA) is widely recognized as an essential component of individual and public health, not only contributing to the prevention of chronic diseases but also enhancing quality of life and psychological well-being [1,2,3,4,5]. Throughout life, participating in PA has been associated with a broad range of benefits, including better cardiovascular health, improved mental health, enhanced cognitive function, and increased social engagement. Therefore, PA is considered a vital element of healthy aging and sustainable public health systems.

However, despite increasing evidence of the health benefits of physical activity (PA), global participation rates remain troublingly low. Recent estimates show that fewer than one in three adults meet the physical activity guidelines set by the World Health Organization [6]. This widespread inactivity has become a major concern for health officials worldwide, as sedentary lifestyles are strongly linked to higher rates of obesity, diabetes, heart disease, and mental health problems, putting a heavy burden on healthcare systems.

Regular physical activity has consistently been linked to improved psychological outcomes, including reductions in anxiety and depression symptoms, better mood and emotional regulation, and higher levels of self-esteem and psychological resilience [7]. People who stay physically active often report greater overall life satisfaction and tend to experience more healthy life years (HLYB) and longer life expectancy at birth (LEB) [1,8,9,10,11,12,13,14]. Additionally, participating in PA during childhood and adolescence is essential for developing motor skills, cognitive functions, and social behaviors, laying a foundation for health that lasts into adulthood [11,12,15,16,17,18].

However, the ability to participate in PA is not just a matter of personal choice or motivation. It is increasingly evident that PA behavior is influenced by a variety of external factors, including socioeconomic status, environmental conditions, cultural norms, and particularly the availability of physical activity options and institutional support systems. Among these factors, the role of local infrastructure—such as parks, sports facilities, and safe walking or cycling routes—and the involvement of local service providers and authorities are especially important.

This study explores how PA-related infrastructure and the involvement of local authorities influence exercise habits and health outcomes across the European Union. While the benefits of PA are well known, there are still significant differences among EU countries in participation rates and access to supportive environments [19]. These differences raise important questions about the structural and institutional factors that either promote or hinder active lifestyles.

This research aims to examine how community-level physical activity opportunities and local government efforts influence two key health metrics: healthy life years and life expectancy at birth. Using a comparative cross-national approach and advanced tools like artificial neural networks and cluster analysis, the study seeks to identify patterns and relationships that can guide public policy. It also addresses a significant gap in existing literature: the lack of integrated models that consider both behavioral and structural aspects of physical activity participation.

The study views physical activity not only as a personal health behavior but also as a socially and institutionally influenced practice, which depends on the interaction between individual agency and systemic support. Understanding this dynamic is essential for developing targeted, effective public health strategies to increase physical activity and reduce health inequalities across the EU.

## 2. Theoretical Background and Hypotheses

### 2.1. Physical Activity and Its Impact on Health and Quality of Life

Although the physical and mental health benefits of physical activity (PA) are well documented, actual participation in PA varies greatly among different population groups. It is influenced by a complex combination of social, economic, and environmental factors. This variation is important for creating inclusive and effective health promotion policies at both local and national levels [1].

Recent studies highlight the positive impact of PA on older adults’ cognitive and physical health, as well as its role in reducing dementia risk and supporting independence [20,21,22,23]. Kiełtyka-Słowik et al. [24] discovered that among individuals aged 60–89, higher PA levels were strongly associated with a better quality of life, emphasizing the importance of remaining active in later years. Similarly, Hao et al. [25] observed that PA was positively related to health-related quality of life across different age groups, with older adults experiencing the greatest benefits.

Additionally, individuals with chronic conditions like diabetes or cardiovascular diseases experience improved health outcomes, such as reduced stress levels and better symptom management, when they engage in regular physical activity [26,27,28,29]. Moreover, physical activity has been shown to increase life expectancy without cancer, with following recommended activity levels associated with nearly two extra years of cancer-free life [30].

Gender-based differences in PA participation are also well established: men are generally more physically active than women, often motivated by competitive and performance-related goals, while women tend to value PA for its emotional and appearance-related benefits [31,32]. These findings highlight the need for tailored strategies that address the specific motivations and barriers faced by different demographic groups. Swartz [33] pointed out that middle-aged women who started or maintained regular exercise by age 55 experienced better physical health outcomes, indicating that targeted interventions could be effective.

Besides biological and psychological factors, PA is deeply rooted in broader socioeconomic structures. People from lower-income backgrounds report lower physical activity levels, often because they lack access to facilities, face unsafe environments, or have limited time due to financial struggles [34]. Education level is another vital factor: those with a higher education are more likely to participate in regular PA and understand its long-term health benefits [15,16,35,36]. Additionally, Gregory [37] showed that maintaining a healthy lifestyle, including consistent PA, could counteract genetic predispositions by over 60% and might add up to five years to one’s lifespan, emphasizing the strong influence of lifestyle choices.

Environmental and contextual factors, such as the built environment, urban planning, and local infrastructure, also significantly influence PA engagement [38]. Access to parks, walking trails, and recreational facilities can notably boost participation rates, mainly when supported by local government efforts and public investment in active living programs [39]. A large-scale study by Althoff et al. [40] showed that moving to more walkable neighborhoods resulted in sustained increases in PA levels, highlighting the importance of urban design in promoting health.

Finally, perceived quality of life is closely linked to PA habits: individuals who engage in regular exercise often report better physical, emotional, and social well-being than those who are inactive [10,41]. These perceptions are influenced by personal experience and broader societal factors, making it crucial to consider both individual and systemic determinants in analyzing PA outcomes. Li et al. [42] found that depression mediates the relationship between PA and health-related quality of life in older adults, indicating that PA may indirectly improve quality of life by reducing depressive symptoms.

In this study, we propose a first hypothesis (H1) grounded in preliminary observations and the existing literature, which highlights a connection between variables:

**Hypothesis** **H1.** *The frequency of participation in PA is significantly positively associated with healthy life years (HLYB) and life expectancy at birth (LEB) across European Union countries.*

Higher activity or participation in sports is believed to be connected to more years of healthy life and a longer life expectancy. In contrast, lower levels of PA are linked to poorer public health outcomes [14].

### 2.2. Physical Activity and Opportunities for Sports and Exercise

Numerous studies have demonstrated that moderate to vigorous physical activity (PA) is closely associated with an increased sense of overall well-being, including environmental, social, mental, and physical dimensions [10,43,44,45,46,47]. Recent research continues to support these findings, highlighting that higher levels of PA contribute to better health-related quality of life across various age groups [24,25].

Social context and residential environment greatly influence PA behaviors. Lee and Cubbin [48] and Ominyi and Clifton [49] emphasize that small, socially cohesive communities show higher participation rates in physical activity, especially among elderly adults [50]. Better sports facilities in urban areas improve access to recreational options, enhancing individuals’ quality of life [51]. Additionally, the interaction between social and built environments plays a key role in shaping PA levels, with socioeconomic status and urban planning significantly affecting access to physical activity opportunities [52].

The availability and quality of infrastructure for PA greatly affect population behaviors related to sports and exercise [53,54]. People living near parks, bike paths, sports fields, and recreational facilities are more likely to adopt active lifestyles. These spaces provide concrete opportunities for activity and foster a social environment that promotes interaction and support [55,56]. Recent systematic reviews indicate that urban interventions, like park upgrades and adding exercise equipment, have a positive effect on physical activity levels [57]. Furthermore, greenway projects have been shown to effectively boost PA, highlighting the importance of integrating natural features into urban planning [58].

Local providers of sports services, including fitness centers, sports clubs, and community associations, play a crucial role in encouraging active lifestyles [59,60]. Programs organized by these organizations, from yoga classes and group training sessions to sports events, attract a wide variety of participants and can foster a sense of belonging and motivation [60,61]. Collaborations between local providers and public authorities can further help develop accessible and inclusive programs that serve entire communities [62].

Local authorities play a key role in creating environments that encourage physical activity (PA). Investing in sports infrastructure and educational programs can greatly impact public health [63]. Authorities can support initiatives that promote PA for all age groups, from children to seniors [1]. For instance, subsidizing gym memberships or hosting free sports events can help boost community participation [60]. Evidence indicates that municipal resources dedicated to physical activity facilities, such as pedestrian and bike paths, parks, and sports arenas, can increase adult physical activity. However, the outcomes may depend on how effectively these are implemented and how engaged the community is [64].

Community programs promoting active lifestyles are especially valuable in disadvantaged areas, where they can significantly improve health outcomes and quality of life [65]. Such initiatives lower the costs linked to treating sedentary-related illnesses and support increased longevity [66]. These programs can help reduce health disparities by enabling all community members to benefit from physical activity [67]. Additionally, public policies focused on increasing physical activity and decreasing sedentary behavior have been recognized as effective strategies to improve public health outcomes [68].

The second hypothesis in this study (H2) explores the connections between the availability of PA opportunities in local areas, the involvement of local authorities, and the frequency of participation in sports and exercise.

**Hypothesis** **H2.** *Hypothesis 2 suggests that the availability of physical activity opportunities in a community and local providers’ support for exercise programs are significantly linked to how often people participate in sports and exercise. Likewise, lower efforts by local authorities to promote physical activity are expected to be associated with a decreased involvement in sports and exercise activities.*

Higher levels of accessible infrastructure and proactive local governance are believed to be linked to increased participation in PA, resulting in better public health outcomes.

## 3. Materials and Methods

### 3.1. Research Design

This study uses a comprehensive methodological framework to analyze the relationships between physical activity (PA) opportunities, local government involvement, and health outcomes across European Union member states. Based on a macro-level analytical approach, the research combines harmonized and cross-nationally comparable data from the Eurobarometer survey [19] and Eurostat database [69]. These sources provide extensive indicators related to PA participation, perceptions of accessibility to PA infrastructure, institutional support, life expectancy, and healthy life years (HLYB).

Two analytical techniques were used to examine the complex and interconnected nature of these variables: artificial neural network (ANN) analysis and cluster analysis. ANN analysis was selected for its ability to detect non-linear patterns and uncover hidden relationships that traditional statistical models often missed. This approach is particularly suitable for social and behavioral data, where interactions among personal, institutional, and environmental factors rarely follow straightforward paths. The neural network was built using a multilayer perceptron with one hidden layer. Multiple training cycles were performed to ensure the model’s stability and reduce the risk of overfitting. The analysis is based on cross-sectional data from 2022, collected through the European Commission and Eurostat, covering all EU member states.

Meanwhile, cluster analysis was used to incorporate a spatial and structural perspective into the findings. This method groups countries into clusters based on shared traits in PA behaviors, perceived accessibility to infrastructure, and public health outcomes. The resulting typologies help identify macro-regional trends, offering valuable context for understanding national results within broader EU-level dynamics and guiding targeted policy actions.

### 3.2. Literature Review Methodology

To ensure the relevance and quality of the reviewed literature, we applied clearly defined inclusion and exclusion criteria. Articles were included if they met the following conditions: they were peer-reviewed journal publications; focused on physical activity and its association with public health outcomes, governance, or infrastructure; were published between 2010 and 2024; and were written in English. Studies had to present either empirical findings or theoretical insights relevant to the European context or comparable international settings.

Exclusion criteria included non-academic sources such as editorials, commentaries, or gray literature; studies not directly related to the research topic or conceptual framework; and articles without full-text access.

The literature search was performed across major academic databases, including Scopus, Web of Science, Google Scholar, and PubMed, using combinations of keywords such as “physical activity,” “public health,” “life expectancy,” “local infrastructure,” “community engagement,” and “European Union.” The initial pool of studies was screened based on titles and abstracts, followed by a full-text review of the articles that met the eligibility criteria.

Key data from the selected studies were extracted and thematically synthesized. Themes were identified through an inductive analysis approach, enabling the recognition of common patterns and differences in how environmental and institutional factors influenced physical activity. These themes then informed the development of the conceptual framework presented in this paper.

### 3.3. Selected Variables

The selection of variables in this study was guided by their theoretical relevance, data quality, and consistent availability across all EU member states. The goal was to build an integrated dataset that captured both the behavioral aspects of physical activity (PA) and the broader environmental and institutional contexts in which these behaviors took place. Ultimately, eight variables were included and categorized into three themes: (1) frequency of physical activity participation, (2) perceived environmental and institutional support, and (3) health outcomes at the population level.

Physical activity engagement was assessed using self-reported data from the 2022 Eurobarometer survey [19]. Respondents indicated how often they exercised or played sports, and their answers were grouped into four frequency levels: regularly, with some regularity, seldom, and never. These categories helped us evaluate the range of PA engagement across different populations consistently.

To assess physical activity–related opportunities, we used three perceptual indicators from the Eurobarometer. The first measured whether respondents felt their local area provided adequate opportunities for physical activity, such as access to parks or walking paths. The second evaluated the perceived availability of local providers, gyms, sports clubs, or community organizations that facilitated or organized PA activities. The third examined the perceived involvement of local authorities in promoting active lifestyles. This last item was assessed based on responses to the question of whether citizens believed the local public authorities made sufficient efforts to promote physical activity. Each of these items was reported as a percentage of positive responses at the national level.

It is essential to recognize that these measures are based on subjective assessments rather than objective infrastructure audits or content analysis of policy documents. However, similar methods have been used in previous studies with Eurobarometer data to evaluate health perceptions at the population level. Although no additional validation studies were conducted in this research, internal consistency checks and demographic benchmarking were used to improve reliability. This approach follows standard practices in comparative health behavior research, especially when direct measurement across countries is not feasible.

The health outcome variables, life expectancy at birth and healthy life years, were obtained from Eurostat [69], providing consistent, harmonized indicators of population health. These outcomes were used to examine long-term relationships between PA behaviors and overall public health trends.

By combining self-reported behavioral data with perception-based environmental indicators and validated health metrics, the study aims to provide a comprehensive overview of how institutional and infrastructural factors influence physical activity engagement and health outcomes across the EU.

Through this methodological design and the variables detailed in Table 1, the study aims to develop a nuanced understanding of how infrastructure, institutional engagement, and perceptions of opportunity interact to influence physical activity behaviors and, ultimately, public health outcomes across EU member states.

### 3.4. Research Methods

The empirical study utilizes two methods to conduct a comprehensive and meaningful analysis, each contributing to a more detailed understanding of the relationships between PA opportunities, local authority involvement, and health outcomes. All analyses were carried out using IBM SPSS (version 27).

To capture the intricate, non-linear interactions among variables that traditional statistical models may not fully detect, we employed an artificial neural network (ANN) approach. Given the complexity of health-related behaviors and their multifactorial nature, ANNs offer a valuable tool for analyzing multidimensional data where variables often influence one another in non-obvious or non-linear ways [70].

For this study, we used a Multilayer Perceptron (MLP) model, a popular ANN architecture known for its effectiveness in predictive modeling. The model was trained using the backpropagation algorithm, which repeatedly adjusts the model’s internal parameters to minimize prediction errors. Structurally, the neural network consists of three main layers: an input layer that receives key predictors such as physical activity participation, infrastructure availability, and local authority involvement; a hidden layer that learns complex patterns from the input data; and an output layer that predicts health outcomes—specifically healthy life years and life expectancy.

Mathematically, the relationship within a neural network can be described by the following formulation (1):

(1) $$y=\varphi (\sum_{i=1}^{n}w_{i}x_{i}+b)=\varphi (W^{T}X+b)$$

In this equation, *x* sub *i* represents the input variables (e.g., survey responses or indicators), *w* sub *i* denotes the weights assigned to each input, and *b* is the bias term. These parameters are adjusted during training to optimize prediction accuracy. The function script φ, known as the activation function, introduces non-linearity into the model, allowing it to approximate more complex relationships.

In our model, we employed a sigmoid activation function, which maps input values to an output range between 0 and 1 (2):

(2) $$f\left(n\right)=\frac{1}{1+e^{-n}}$$

*n*—input variables;
*f*(*n*)—output variables.

This function is handy for modeling probabilities and health-related classifications because it provides smooth, differentiable transitions between output values and helps avoid extreme outputs.

**Figure 1:**
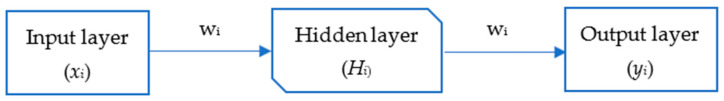
MLP model.

Figure 1 illustrates the key components of the network, including the input variables, the hidden layer that processes information, and the output layer that generates predictions for health-related outcomes.

The cluster analysis used SPSS’s hierarchical clustering procedures to systematically identify natural groupings among EU countries based on their physical activity patterns and health outcome profiles. This multivariate technique offers insights into targeted policymaking by revealing which nations face similar challenges and opportunities in promoting population health through physical activity initiatives. We used squared Euclidean distance for the distance matrix calculation, as this measure effectively captures the magnitude and pattern of differences between country profiles across multiple dimensions. The clustering algorithm proceeded with the average linkage (between groups) method, specifically chosen for its optimal balance between the sensitivity of single linkage and the compactness of complete linkage approaches [71,72]. This method calculates the mean distance between all possible pairs of observations across two clusters, making it exceptionally robust to outliers while maintaining meaningful cluster boundaries. The linkage distance determines cluster membership (3):

(3) $$d_{ij}=\frac{1}{kl}\sum_{i=1}^{k}\sum_{j=1}^{l}d(X_{i},Y_{j})$$

$X_{1,}X_{2,},\ldots ,X_{k}$—observations from Cluster 1,
$Y_{1,}Y_{2,},\ldots ,Y_{l}$—observations from Cluster 2,
*d*(*X*,*Y*)—distance between a subject with observation vector x and a subject with observation vector,
*k*,*l*—cases.

Combining these two methods creates a strong analytical framework that identifies complex variable relationships and important country groupings, providing thorough insights for developing public health policies.

## 4. Results

**Figure 2:**
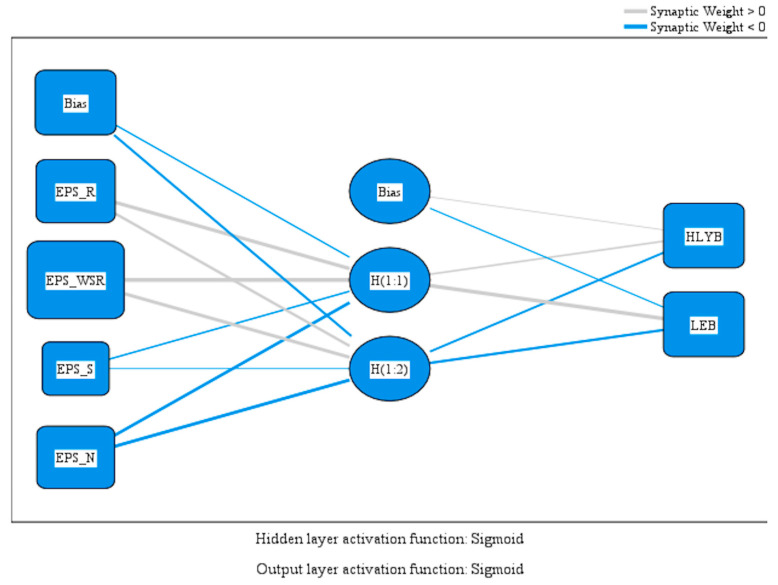
MLP model predicting healthy life years and life expectancy at birth. Source: authors’ design using SPSS v27.0 (SPSS Inc., Chicago, IL, USA).

The first hypothesis (H1) proposes that higher frequencies of physical activity (PA) are positively associated with more healthy life years and longer life expectancies at birth across European Union countries. To test this, the study employed a Multilayer Perceptron (MLP) model designed to capture complex relationships between variables, featuring a hidden layer with two neurons that serve as internal computational nodes emerging from training, representing active and inactive behavioral patterns based on their weight distributions. Figure 2 displays the MLP model’s output, estimating the impact of physical activity participation and institutional factors on two key public health indicators: healthy life years and life expectancy at birth. The visualization highlights the relative importance of each predictor in shaping population health. From a policy perspective, the findings suggest that consistent physical activity engagement, supported by ongoing public-sector efforts, is strongly linked to improved long-term health outcomes. This underscores the potential of integrated policy strategies that combine behavioral promotion with structural support.

Table 2 displays the expected values of the perceptron’s model variables.

The model’s results emphasize the unique impact of various physical activity (PA) behaviors on public health outcomes, demonstrating the practical importance of regular movement for improving population health.

Among the input variables, regular physical activity (EPS_R) showed a strong positive impact (2.366) in the model, highlighting the connection between consistent PA and improved health, especially in increasing healthy life years (HLYB). This supports the idea of regular PA as a key public health strategy, emphasizing its potential to reduce chronic disease rates and extend lifespans.

Even more noteworthy is the influence of moderate regular exercise (EPS_WSR), which has the highest positive weight (4.282). This finding suggests that consistent but not necessarily intense physical activity can offer the greatest health benefits. The significance of this variable highlights the importance of encouraging moderate-intensity activity, which is accessible to a broader group of people, as an effective and inclusive strategy for public health promotion. These results provide valuable guidance for policymakers seeking to develop realistic and equitable health initiatives.

In contrast, infrequent exercise (EPS_S) and especially no exercise (EPS_N) had negative weights in the model, with EPS_N showing the strongest negative correlation. This result aligns with previous research and confirms the significant health risks associated with sedentary lifestyles. Populations with little or no physical activity are more likely to have shorter health spans and lower life expectancies, emphasizing the importance of tackling inactivity as a public health issue.

Regarding the dependent variables, both healthy life years at birth (HLYB) and life expectancy at birth (LEB) were positively linked to the H(1:1) neuron, especially LEB (weight = 2.395). Conversely, the H(1:2) neuron consistently showed negative weights. These findings further support the hypothesis that physical activity (PA) positively impacts public health outcomes and that inactivity leads to negative health trajectories.

Overall, the findings strongly support Hypothesis H1 by showing significant links between PA frequency and public health outcomes. Additionally, the results have important policy implications: health improvements are possible not only through high-intensity PA but also through moderate, sustainable activity levels. This broadens the options for creating inclusive and scalable public health strategies.

**Figure 3:**
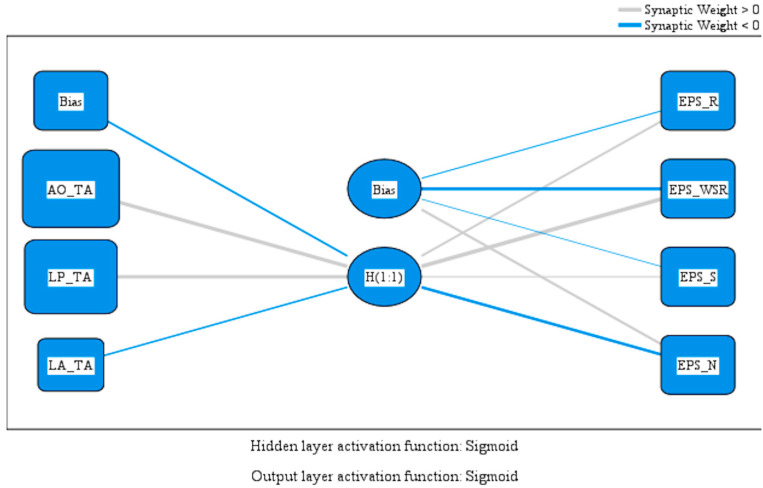
MLP model assessing access to physical activity opportunities. Source: authors’ design using SPSS v27.0 (SPSS Inc., Chicago, IL, USA).

Hypothesis H2 proposes that the availability of PA opportunities and support from local service providers are closely connected to participation in sports and exercise. Conversely, areas where local authorities show limited effort in promoting PA tend to have lower levels of physical activity. The Multilayer Perceptron (MLP) model used in this study confirms this hypothesis, illustrating how contextual and institutional factors influence individual behavior. Figure 3 displays the predictive weights of variables affecting the availability of physical activity opportunities across EU member states. The MLP model emphasizes key structural and institutional factors that impact accessibility, such as local authority support and infrastructure. The findings highlight the importance of effective governance and strategic investment in creating supportive environments. Policymakers can interpret these insights as a call to improve cross-sector coordination and ensure equitable access to physical activity, particularly in underserved regions.

Table 3 displays the expected values of the perceptron’s model variables.

An analysis of the model parameters provides valuable insights into how environmental and institutional factors affect physical activity (PA) participation across populations.

The variable representing opportunities for physical activity has a strong positive influence (3.179) on the hidden neuron H(1:1). This indicates that environments with well-developed infrastructure, such as parks, sports fields, and recreational facilities, greatly encourage active behaviors. In summary, when the public regularly has access to suitable spaces for exercise, the chances of consistently engaging in sports and physical activity rise significantly.

Similarly, the variable indicating the presence of local providers who support and organize exercise-related programs also shows a positive impact (3.112). This highlights the important role of local actors, such as fitness centers, sports clubs, and community organizations, in promoting population-wide PA. Initiatives like sports leagues, running events, and educational campaigns about the benefits of exercise help build a culture of activity and participation within communities.

In contrast, the variable representing the absence of involvement by local authorities has a negative weight (−1.342). A lack of public awareness campaigns, insufficient investment in sports infrastructure, and limited support for active lifestyle programs seem to discourage participation in PA. This negative impact partially offsets the benefits provided by infrastructure and provider support, emphasizing the need for an integrated, multisectoral strategy where local governments work with community stakeholders to promote active living.

At the hidden layer level, neuron H(1:1) plays a key role in predicting PA behaviors, especially regarding available opportunities. Its weighted contributions to the output variables are notably positive: regular exercise (EPS_R: 1.613), moderate regular exercise (EPS_WSR: 3.609), and even infrequent exercise (EPS_S: 1.263). These values indicate that the presence of opportunities is strongly associated with an increase in active behaviors, particularly in encouraging consistency and routine physical activity.

Conversely, the model assigns a negative weight (−2.552) from H(1:1) to EPS_N, indicating no physical activity. This result suggests that environments enriched with opportunities and community support decrease the likelihood of populations staying completely sedentary. However, the hidden layer’s positive bias term for EPS_N (1.658) shows a latent tendency toward inactivity when supportive infrastructure and initiatives are lacking. This insight supports the idea that, without external motivators, populations may tend to adopt more sedentary lifestyles.

Analyzing the importance values in the Multilayer Perceptron (MLP) model clarifies the relative influence of each independent variable on predicting PA participation. The results strongly support Hypothesis H2, showing that both environmental opportunities and the presence of engaged local providers are positively and significantly linked to higher levels of physical activity. Conversely, limited involvement by local authorities is associated with lower activity rates, suggesting a potential institutional barrier to wider public participation in PA.

**Figure 4:**
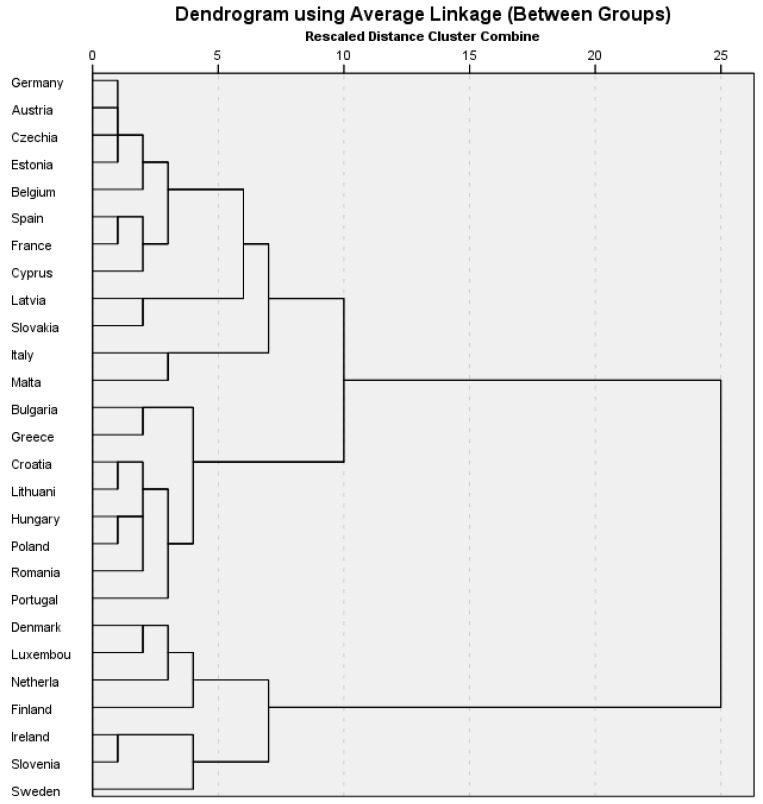
Cluster groupings of EU countries based on physical activity indicators and health outcomes. Source: authors’ design using SPSS v27.0 (SPSS Inc., Chicago, IL, USA).

Complementing the neural network analysis, the cluster analysis of EU member states, based on life expectancy, healthy life years, PA behaviors, and access to opportunities, reveals a complex and nuanced landscape. Figure 4 shows the three distinct clusters of EU member states identified through neural network and cluster analysis. Each group reflects a unique combination of physical activity levels, access to infrastructure, and institutional engagement, which together influence national health outcomes such as healthy life years and life expectancy. This perspective highlights the connection between behavioral, structural, and policy factors, emphasizing the need for context-sensitive interventions that address regional differences and promote health equity across Europe.

The cluster analysis reveals distinct groupings among EU member states, highlighting significant differences not only in physical activity behaviors and health outcomes but also in the institutional and environmental factors that affect them (Table A1, Appendix A). These differences suggest that a one-size-fits-all approach to promoting physical activity (PA) is unlikely to be effective. Instead, interventions should be tailored to each cluster’s specific combination of resources, behaviors, and governance dynamics.

Countries in Cluster 1, including Denmark, the Netherlands, Finland, and Sweden, display the most favorable profiles, with high levels of regular and moderately frequent physical activity (PA), long life expectancies, and many healthy life years. These countries have well-developed PA infrastructure and strong support from community-level service providers. Interestingly, the influence of local government appears less noticeable, possibly because the existing physical and cultural environment already promotes high engagement in PA. In such settings, policy efforts might focus on maintaining quality standards, reducing social inequalities, and increasing access for underrepresented or vulnerable groups, rather than expanding basic infrastructure. These systems could serve as models for improving public health strategies elsewhere.

Cluster 2, which includes countries such as Germany, France, Spain, and Italy, occupies a middle ground. These nations have above-average health indicators, but PA behaviors are less consistent, and access to infrastructure varies significantly. The most notable feature of this cluster is the inconsistency in local authority engagement, which ranges from proactive to minimal. This variability indicates that policy coherence and sustained public-sector leadership may be more influential here than infrastructure alone. In this group, tailored interventions should aim to align local policies with national health goals, build cross-sectoral partnerships, and ensure that available infrastructure is matched with culturally relevant programs and long-term community engagement strategies.

Cluster 3, which includes Bulgaria, Greece, Romania, and Hungary, faces the most urgent challenges. These countries report the lowest levels of PA and the highest rates of inactivity, with limited infrastructure and weak engagement from local authorities and service providers. The strong negative correlation between low institutional support and poor health outcomes in this group indicates that the lack of public-sector leadership is not just a missed chance but may actively contribute to health inequity. In such situations, interventions should focus on building basic infrastructure, investing targeted resources in disadvantaged areas, and establishing essential governance mechanisms to coordinate PA promotion efforts. Without a visible public-sector presence, community or private initiatives are unlikely to reach scale or ensure long-term sustainability.

The three clusters emphasize the need for tailored, context-aware policy responses. While Cluster 1 offers models for consolidation and quality improvement, Clusters 2 and 3 emphasize the importance of greater strategic alignment, institutional capacity building, and targeted investments. These findings support both study hypotheses (H1 and H2) and underline that effective public health policy must incorporate structural, behavioral, and institutional elements to ensure equitable access to active living environments across Europe.

## 5. Discussion

This study emphasizes how various local and institutional arrangements influence patterns of PA across the European Union and their connections to public health outcomes. By combining artificial neural network modeling with cluster analysis, the results demonstrate that higher engagement in PA, especially when sustained at a moderate level, is strongly linked to increased healthy life years and longer life expectancies. These findings support both study hypotheses: that regular PA correlates with better health (H1), and that supportive environments and institutional support are key factors driving participation (H2). This relationship develops not as a simple linear path but as a result of interconnected social, infrastructural, and governance-related elements [65].

The neural network model supports the idea that moderate, sustained activity—not necessarily intense or performance-oriented—is most closely linked to public health benefits. This finding aligns with a growing trend in the literature emphasizing inclusivity and feasibility over athleticism [1,10,66,73]. It also echoes the Health Belief Model, which suggests that health behaviors are influenced by perceived accessibility and benefits rather than intensity [74]. Promoting PA as a routine and achievable activity integrated into daily life increases its adoption across different ages, genders, and socioeconomic backgrounds.

Gender differences in how people perceive and use physical activity resources reflect broader behavioral and social patterns. Research shows that men tend to prefer structured or competitive sports, while women often favor informal or socially connected activities. These choices are influenced by factors such as perceived safety, time constraints, and caregiving responsibilities, which can limit women’s access to available infrastructure [75]. These insights highlight the importance of gender-sensitive planning to create physical activity environments that are equitable and meet diverse needs.

Additionally, the findings support a broader health perspective that includes mental and emotional well-being. Interventions such as greenway infrastructure and low-threshold activity programs have been shown to reduce psychological distress and improve subjective well-being, especially when integrated into community settings [58]. Age-specific differences, as demonstrated by Hao et al. [25], further highlight the need for PA strategies tailored to different life stages. Overall, these results advocate for a flexible and responsive PA promotion paradigm that considers real-life experiences.

Notably, the study highlights regions—such as Bulgaria and Romania—where low PA participation is linked to weak institutional support and limited infrastructure. Here, health disparities are worsened not just by individual behaviors but also by environmental and systemic deprivation [34]. This supports insights from the Ecological Model, which views health behavior as the result of interactions across individual, interpersonal, community, and policy levels [38]. Interventions that focus only on individual motivation are likely to fall short unless broader conditions—such as access, affordability, and cultural relevance—are addressed simultaneously.

The analysis further clarifies that infrastructure, while essential, is not sufficient. Access to parks, gyms, or recreational facilities must be supported by culturally meaningful programming, institutional trust, and efforts to reduce social fragmentation [53,55,59,76]. These findings reinforce a systems-thinking approach in health promotion—one that considers physical activity behavior as emerging from complex social ecosystems rather than static resources [39,54].

Institutional engagement plays a crucial role in motivating community action. Local governments do more than just oversee; their involvement legitimizes public health initiatives, encourages social norms around activity, and fosters trust among residents [77,78,79,80,81,82,83]. In our model, active participation from local authorities was strongly linked to higher levels of physical activity and more healthy life years, supporting the Ecological Model’s focus on structural and relational factors that enhance health.

These patterns align with existing research. Høyer-Kruse et al. [52] emphasize how socioeconomic factors and urban planning influence access to physical activity spaces, supporting the need for inclusive and equity-focused infrastructure design. Similarly, Zhang et al. [57] demonstrate that improvements to built environments—such as safer parks or better bike paths—can greatly increase physical activity if they are accessible, visually appealing, and address community needs.

Overall, the findings support a shift in how we view PA as a socially connected behavior rather than just an individual duty. The study adds to an increasing body of research that stresses the importance of comprehensive, multisectoral strategies that blend behavioral theory with real investment in infrastructure and governance. For these strategies to be effective and fair, they need to address not only the physical factors that promote activity but also the social trust and institutional leadership that maintain it [48,49].

### 5.1. Theoretical and Practical Insights on Physical Activity Determinants

The findings of this study support the growing understanding that physical activity patterns are influenced by both individual circumstances and the broader environment. The idea that health behaviors are driven solely by personal willpower or knowledge neglects the roles of physical accessibility, community norms, and policy signals. In this context, the study advances existing research by introducing a more comprehensive theoretical framework in which the frequency of physical activity results from the interaction among opportunity, governance, and lived experience.

In environments where residents consistently have access to quality infrastructure, such as recreational spaces, fitness facilities, and well-maintained urban paths, there is a higher chance that physical activity (PA) will become part of daily life. However, this effect is amplified when such infrastructure is backed by visible institutional support. The interaction between these elements seems essential; environments that are physically equipped but lack programming, outreach, or ongoing funding may struggle to change behaviors, especially in socioeconomically disadvantaged areas where additional barriers exist.

Notably, the study shows that institutional presence acts as a behavioral enhancer. Local authorities and community service providers can increase the effectiveness of physical spaces by developing inclusive programs, promoting participation, and tackling psychosocial barriers. This interaction emphasizes the need for improved theoretical models that consider individual behaviors within both material and symbolic frameworks. The existence of authority-led initiatives may indicate that activity is recognized and supported, which can be especially vital in communities with lower self-efficacy or fewer resources. These dynamics reveal that physical activity is as much a social and political issue as it is a health concern.

### 5.2. Policy and Community-Level Strategies for Promoting Physical Activity

In policy terms, the study affirms the need for integrated, equity-focused strategies to encourage physical activity. Building infrastructure alone is insufficient; health-promoting environments must also be socially inclusive, financially accessible, and culturally responsive. Investments in recreational spaces, for example, should align with the daily routines and perceived needs of local communities, especially in underserved areas where historical neglect has created mistrust and disengagement. Targeted investments in infrastructure and programs within marginalized communities are not just about redistribution; they are a health necessity to close gaps in well-being and longevity.

The findings also highlight the key role of community involvement in promoting active lifestyles. While infrastructure provides the setting, community-based initiatives, local sports programs, and culturally relevant events are what motivate participation and maintain engagement. Effective strategies stem from partnerships where local governments, nonprofits, schools, and private organizations collaborate to develop accessible and meaningful interventions. This teamwork increases participation and ensures that programs reflect the real experiences and hopes of the people they aim to serve.

Furthermore, the analysis emphasizes the need for long-term policy integration. Promoting PA shouldn’t be viewed as a standalone goal; it must be embedded into wider health promotion strategies, educational initiatives, transportation planning, and urban design. Environments that make movement a normal part of daily life, rather than just encouraging it, hold the greatest potential for lasting impact. When people have opportunities to be active at their schools, workplaces, and neighborhoods, activity becomes part of the social fabric instead of an isolated choice.

The variation across EU member states highlights the importance of tailoring interventions to local conditions. Some countries might benefit from expanding successful models, while others require fundamental investments in infrastructure and institutional capacity. Recognizing this diversity is essential for promoting inclusive and effective health initiatives. Only by adapting strategies to each area’s specific socioeconomic, cultural, and political contexts can policies achieve both a wide-reaching impact and meaningful engagement.

Overall, this study confirms that physical activity is not just a personal effort or the result of isolated interventions. Instead, it results from complex interactions among material, institutional, and symbolic factors that need to be understood together. Building these connections offers a promising path toward healthier, more equitable societies.

### 5.3. Limitations and Further Research

While this study offers valuable insights into the relationship between physical activity (PA), infrastructure, and health outcomes across the European Union, it is important to acknowledge several limitations that could influence how the findings are interpreted and their overall relevance.

First, although using secondary, cross-sectional, and self-reported data allows for standardized cross-country comparisons, it also introduces inherent limitations—such as potential reporting bias, recall inaccuracies, and the inability to establish causality—that we aimed to mitigate through data triangulation using Eurobarometer and Eurostat sources, internal consistency checks, and artificial neural network modeling. We also recognize that aggregating data at the national level can overlook regional differences, a gap partially addressed through cluster analysis but one that requires more detailed, localized studies in future research. Longitudinal data would be more suitable for examining how these dynamics evolve over time.

Second, the dataset is limited to EU member states, reflecting their unique cultural, socioeconomic, and policy contexts. As a result, the findings may not apply to other regions around the world where different institutional structures, economic conditions, or cultural norms could significantly affect PA behaviors and public health outcomes.

Third, although the study concentrates on key variables such as PA patterns, accessibility, and institutional support, it does not include several other well-established determinants of health. These include socioeconomic indicators like GDP per capita, healthcare system capacity, education levels, and broader social determinants. The omission of these variables, even though it aims to keep the analysis clear, may restrict the overall scope of the conclusions. Therefore, the robustness of the model could be enhanced by adding a wider array of predictors.

Furthermore, the current analysis operates at the population level, which limits its ability to capture the nuanced complexity of individual behaviors. Personal motivations, perceived barriers, and psychological factors influencing PA participation were not directly assessed. This macro-level perspective, while helpful in identifying regional patterns, may overlook disparities within countries or dynamics specific to particular subgroups.

Future research should address these limitations by including a wider range of variables to create a more comprehensive understanding of what influences health outcomes and life expectancies. Specifically, adding economic indicators, healthcare access data, and social inequality measures could provide deeper insights into the complex factors affecting public health. Additionally, examining how these factors interact with PA patterns might help explain differences in health outcomes both across and within countries.

Expanding the analysis to a longitudinal framework would also be helpful. Tracking PA behaviors and public health indicators over time would provide stronger evidence of causal relationships and enable an assessment of the long-term effects of public health interventions. Another key area for future research involves exploring individual-level factors, such as perceived health status, motivation to engage in PA, and barriers to participation. Understanding these personal aspects would complement the current macro-level approach and offer a more complete picture of behavior development.

Comparative studies involving regions outside the EU could enhance the generalizability of the findings. Analyzing differences in public policies, cultural attitudes, and environmental factors would show how much institutional frameworks affect PA engagement worldwide.

Future research should explore the expanding role of digital technologies, such as mobile health apps, wearable devices, and online fitness platforms, in encouraging physical activity. These tools are increasingly influencing how people engage with PA opportunities and may serve as key mechanisms for supporting active lifestyles, especially in under-resourced or digitally connected populations.

In summary, while the current study advances understanding of PA-related health determinants within the EU, expanding the scope of analysis in future research will help produce more detailed and practical insights for policymakers and public health professionals.

## 6. Conclusions

This study aimed to examine how locally available opportunities for physical activity, combined with the involvement of local authorities and service providers, influence participation in exercise and impact broader health outcomes across the European Union. The analysis revealed a compelling narrative that connects environmental conditions and governance structures with how individuals engage in movement and ultimately experience well-being throughout their lives.

By framing physical activity within a conceptual structure that includes social, institutional, and behavioral factors, the study provides a more nuanced understanding of what influences active living. The findings support the idea that health behaviors do not happen in isolation. They are shaped—or limited—by support networks, resource access, and infrastructure. When communities have inclusive spaces, visible support from local organizations, and programs that reflect their social realities, physical activity becomes not just an individual goal but a shared cultural norm.

The practical implications of this are significant. Policymakers and practitioners are reminded that encouraging movement extends beyond messaging campaigns; it demands a strong investment in people and communities. Giving priority to fair access to recreational infrastructure, building collaborations between public agencies and community groups, and integrating physical activity into broader health and urban development plans may be key in bridging the gap between awareness and action.

At the same time, this research encourages further exploration. Much remains to be understood about how cultural values, economic disparities, and life-course transitions affect physical activity behaviors across various national contexts. Future studies could improve this discussion by employing longitudinal methods, including psychosocial variables, and comparing regional patterns outside the EU. Digital tools, urban innovations, and participatory planning models also hold the potential to increase access and inclusion in ways that are only beginning to be explored.

Bringing these threads together, the study not only provides empirical insights but also highlights the urgent need to reframe physical activity as an essential public good. To transition from understanding to action, both EU-level institutions and national governments should prioritize coordinated, equity-focused interventions that incorporate physical activity into broader health, education, urban planning, and social inclusion policies. This requires ongoing public investment, stronger intersectoral collaboration, and policies that address local realities while aligning with EU-wide health goals. Ultimately, building healthier and more active societies depends on making physical activity not only possible but also appealing, accessible, and seamlessly integrated into daily life, so that the decision to move becomes the easiest and most supported choice for all citizens.

## Figures and Tables

**Table 1:** Research variables.

Variable	Dataset	Measures	References
EPS_R	Exercise or play sports—Regularly	Percentage	[19]
EPS_WSR	Exercise or play sports—With some regularity	Percentage	[19]
EPS_S	Exercise or play sports—Seldom	Percentage	[19]
EPS_N	Exercise or play sports—Never	Percentage	[19]
AO_TA	Area opportunities to be physically active	Percentage	[19]
LP_TA	Local provider opportunities to be physically active	Percentage	[19]
LA_TA	Lack of local authority efforts in relation to PA	Percentage	[19]
HLYB	Health expectancy in absolute values at birth	Years	[69]
LEB	Life expectancy in absolute values at birth	Years	[69]

Source: developed by the author based on the European Commission [19] and Eurostat [69].

**Table 2:** Parameters of the MLP model on healthy life years and life expectancy at birth.

Predictor		Predicted				Importance	Normalized Importance
		Hidden Layer 1		Output Layer			
		H(1:1)	H(1:2)	HLYB	LEB		
Input Layer	(Bias)	−0.230	−0.570				
	EPS_R	2.366	1.169			0.233	44.7%
	EPS_WSR	4.282	1.804			0.521	100.0%
	EPS_S	−0.296	−0.112			0.035	6.8%
	EPS_N	−1.689	−1.704			0.211	40.5%
Hidden Layer 1	(Bias)			0.152	−0.177		
	H(1:1)			0.332	2.395		
	H(1:2)			−0.516	−0.952		

Source: developed by the authors using SPSS v.27.0 (SPSS Inc., Chicago, Illinois, United States).

**Table 3:** Parameters of the MLP model on the opportunities offered to the population to be physically active.

Predictor		Predicted					Importance	Normalized Importance
		Hidden Layer 1	Output Layer					
		H(1:1)	EPS_R	EPS_WSR	EPS_S	EPS_N		
Input Layer	(Bias)	−1.583						
	AO_TA	3.179					0.446	100.0%
	LP_TA	3.112					0.405	90.8%
	LA_TA	−1.342					0.148	33.3%
Hidden Layer 1	(Bias)		−0.970	−2.826	−0.934	1.658		
	H(1:1)		1.613	3.609	1.263	−2.552		

Source: developed by the authors using SPSS v.27.0 (SPSS Inc., Chicago, IL, USA).

## Data Availability

Data is available in a publicly accessible repository. https://ec.europa.eu/eurostat/databrowser/view/hlth_hlye__custom_15379604/default/table?lang=en (accessed on 2 February 2025); Special Eurobarometer 525. 2022. Available online: https://europa.eu/eurobarometer/api/deliverable/download/file?deliverableId=83675 (accessed on 2 February 2025).

## Appendix A

**Table A1:** Clusters.

	HLYB	LEB	EPS_R	EPS_WSR	EPS_S	EPS_N	AO_TA	LP_TA	LA_TA
Denmark	55.9	81.3	11	48	21	20	89	87	37
Luxembourg	60.2	83.0	13	50	16	21	87	83	47
Netherlands	58.5	81.7	7	53	15	25	91	91	30
Finland	57.9	81.2	18	53	21	8	84	84	20
Ireland	66.0	82.6	13	41	11	35	82	83	45
Slovenia	66.7	81.3	11	41	23	25	80	74	49
Sweden	66.5	83.1	9	50	29	12	89	86	42
Cluster 1 means	61.7	82.0	11.7	48.0	19.4	20.9	86.0	84.0	38.6
EU means	62.1	79.9	7.6	32.8	19.6	40.0	73.6	70.4	43.6
Germany	61.1	80.7	8	35	25	32	85	81	27
Austria	60.9	81.4	7	35	23	35	78	67	30
Czechia	61.8	79.0	7	37	30	26	77	72	40
Estonia	59.3	78.1	8	34	28	30	78	74	27
Belgium	63.7	81.8	4	39	29	28	83	84	47
Spain	61.2	83.2	11	31	11	47	79	80	50
France	64.4	82.3	8	33	14	45	81	75	31
Cyprus	66.0	81.6	11	29	14	46	64	57	53
Latvia	54.2	74.5	9	30	28	33	66	67	36
Slovakia	57.3	77.0	6	29	22	43	59	50	51
Italy	67.4	82.8	3	31	10	56	65	62	57
Malta	70.2	82.4	7	25	37	31	67	85	66
Cluster 2 means	62.3	80.4	7.4	32.3	22.6	37.7	73.5	71.2	42.9
EU means	62.1	79.9	7.6	32.8	19.6	40.0	73.6	70.4	43.6
Bulgaria	66.7	74.2	4	17	18	61	41	41	46
Greece	67.0	80.8	4	19	9	68	61	54	45
Croatia	60.3	77.7	6	24	30	40	62	58	57
Lithuania	60.3	75.8	9	23	15	53	73	56	37
Hungary	62.6	76.0	4	22	15	59	74	69	48
Poland	62.4	77.2	2	21	12	65	73	62	52
Portugal	59.1	81.8	4	18	5	73	67	67	54
Romania	59.0	75.1	2	18	18	62	53	51	54
Cluster 3 means	62.2	77.3	4.4	20.3	15.3	60.1	63.0	57.3	49.1
EU means	62.1	79.9	7.6	32.8	19.6	40.0	73.6	70.4	43.6
